# Pyroptosis of syncytia formed by fusion of SARS-CoV-2 spike and ACE2-expressing cells

**DOI:** 10.1038/s41421-021-00310-0

**Published:** 2021-08-24

**Authors:** Huabin Ma, Zhoujie Zhu, Huaipeng Lin, Shanshan Wang, Peipei Zhang, Yanguo Li, Long Li, Jinling Wang, Yufen Zhao, Jiahuai Han

**Affiliations:** 1 Institute of Drug Discovery Technology, Ningbo University, Ningbo, Zhejiang China; 2 State Key Laboratory of Cellular Stress Biology, School of Life Sciences, Xiamen University, Xiamen, Fujian China; 3 Qian Xuesen Collaborative Research Center of Astrochemistry and Space Life Sciences, Ningbo University, Ningbo, Zhejiang China; 4 Research Unit of Cellular Stress of CAMS, Cancer Research Center of Xiamen University, Xiang’an Hospital of Xiamen University, School of Medicine, Xiamen University, Xiamen, Fujian China; 5 Department of Emergency, Zhongshan Hospital of Xiamen University, Xiamen, China

**Keywords:** Cell death, Mechanisms of disease

Dear Editor,

Coronavirus Disease 2019 (COVID-19) is an infectious disease associated with systematical multi-organ failure caused by SARS-CoV-2, which mainly infects the lung and upper respiratory system^1,2^. Massive multinucleated syncytia are commonly observed in autopsy of severe COVID-19 patients^3^. It has been reported that the interaction between Spike (S) protein and ACE2 not only mediated the fusion of virus with host cells, but also multinucleated giant cells formation^4–7^. However, the underlying molecular mechanisms of syncytia death are poorly understood.

To better observe the formation of syncytia, we established an in vitro cell-cell fusion system to mimic the fusion of SARS-CoV-2 infected cells with ACE2-expressing cells. The SARS-CoV-2-S-GFP expressing cells were imaged by high-content imaging with confocal laser scanning microscope. We found that SARS-CoV-2-S-GFP mainly appeared on the cell membrane surface^8^, as it co-located with cell membrane marker PLCδ-PH (Supplementary Fig. S1a), but not with nuclear RFP (Supplementary Fig. S1e). In addition, S protein was detected as puncta in the cytoplasm with co-localization of Golgi marker GGA1 (Supplementary Fig. S1b), consistent with report showing that S protein was glycosylated in Golgi apparatus^9^. However, two endosome-related proteins Rab5a and Rab7a did not co-locate with the S protein (Supplementary Fig. S1c and d). Then, the SARS-CoV-2-S-GFP expressing cells (HeLa-S-GFP) were co-cultured with A549 expressing ACE2 cells (A549-PLC-RFP- ACE2) at a 1:1 ratio. Syncytia were observed 4 h later (Fig. 1a), and the cell-cell fusion occurred between the cell membranes because the nuclei were intact (Fig. 1b). In addition, syncytia formation in the co-culture system was dependent on the expression of Spike and ACE2 (Supplementary Fig. S2), and regardless of whether the cells were the same type (A549-S-GFP and A549-ACE2, Supplementary Fig. S3a) or different types (H1299-S-GFP and A549-ACE2, Supplementary Fig. S3b; HeLa-S-GFP and A549-ACE2, Fig. 1a), or even from different species (L929-S-GFP and A549-ACE2, Supplementary Fig. S3c).

**Fig. 1:**
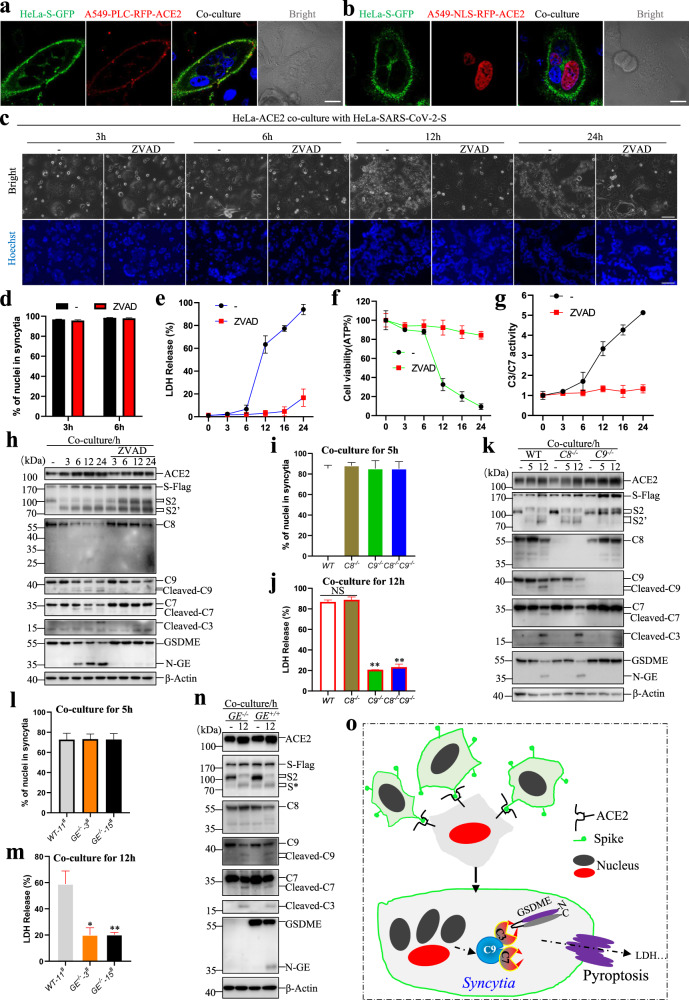
Caspase-9/GSDME trigged pyroptosis of syncytia formed by fusion of SARS-CoV-2 Spike and ACE2-expressing cells. SARS-CoV-2-S-GFP HeLa cells were co-cultured with PLC-RFP-A549-ACE2 cells (**a**), or NLS-RFP-A549-ACE2 cells (**b**) at a 1:1 ratio. Four hours later, image was scanned by LSM780. The nucleus (blue) was stained by Hoechst; Bar, 20 μm. **c** The time-phase of cell-cell fusion progress, HeLa-ACE2 co-culture with HeLa-SARS-CoV-2-S cells, treatment with pan-caspase inhibitor, ZVAD (20 μM) or not; Images were obtained at 3 h, 6 h, 12 h 24 h; the nucleus(blue) was stained by Hoechst; Bar, 100 μm; then the cell-cell fusion was quantified (**d**); the LDH release (**e**), ATP level (**f**), the activity of caspase-3/7 (**g**) were detected at indicated time. Western blot analysis of the cells collected as indicated. ACE2, Flag, caspase-8 (C8), caspase-9 (C9), caspase-7 (C7), Cleaved-caspase-3 (Cleaved-C3), and GSMDE were probed. β-Actin as loading control (**h**, **k** and **n**). The cell-cell fusion was quantified after co-culture for 5 h as indicated (**i** and **l**). LDH release was measured after co-culture for 12 h as indicated (**j** and **m**). The model of SARS-CoV-2 Spike and ACE2 interaction induced syncytia pyroptosis (**o**). The data were shown as means ± SD, *P < 0.05, **P < 0.01, NS non-significance, and all the experiments were replicated more than three times.

Next, we aimed to investigate the fate of syncytia. We found that multiple bubbles were formed on the membrane of syncytia, which tended to rupture at 12 h after co-culture (Supplementary Fig. S3d and e). Then the real-time observation system was used to record the fusing process of SARS-CoV-2-S (HeLa-S) expressing HeLa cells with HeLa-ACE2 cells (Supplementary Movie S1). We found that syncytia were formed upon cell-cell fusion, grew in size steadily, and finally ruptured with LDH release, ATP decrease, and caspase-3/7 activity increase (Fig. 1c-g) as well as IL1β release in the case of THP-1-ACE2 cells co-culture with HeLa-Spike cells (Supplementary Fig. S4). Interestingly, the death was blocked by pan-caspase inhibitor ZVAD, although ZVAD did not affect the syncytia formation. As S protein priming by protease TMPRSS2 or Cathepsin L is essential for cell-cell fusion^7,10^, we explored the expression of S protein under ZVAD treatment. We found that ZVAD did not affect S protein priming because the bands of S2 and S2’ fragment were the same in control and in ZVAD-treated cells, and ZVAD treatment also failed to inhibit the entry of SARS-CoV-2-Spike pseudovirus into HeLa-ACE2 cells, while chloroquine (CQ) inhibited this event (Supplementary Fig. S5a and b). Next, we assessed the activation of molecules related to death. As shown in Fig. 1h, S2 fragment seemed to be modified because of a little “up-shift” in SDS-PAGE gel, and S2’ was moderately induced upon cell-cell fusion. We also found that caspase-9 and caspase-3/7 were cleaved, which generally implies the activation of apoptosis pathway. Interestingly, however, we detected the cleavage of GSDME. It is known that activation of GSDME is mediated by caspase-3^11,12^, and we confirmed the role of caspase in activating GSDME as ZVAD effectively blocked GSDME cleavage. Thus, it can be proposed that syncytia formation led to activation of caspase-9 to caspase-3/7 cascade. The activated caspase-3 cleaved GSDME and released N-GSDME to permeabilize cell membrane to execute syncytia pyroptosis.

To define the death pathway underlying syncytia pyroptosis, we knocked out caspase-8 (*C8*^*−/−*^) or caspase-9 (*C9*^*−/*^^−^) in HeLa cells by CRISPR-Cas9 and expressed ACE2 and SARS-CoV-2-S-Flag in these cells respectively, and then co-cultured these cells in pairs. We found that syncytia formed in *C8*^*−/−*^, *C9*^−^^*/−*^ and *C8*^−^^*/−*^*C9*^*−/−*^ cells, which showed no difference from WT cells (Fig. 1i and Supplementary Fig. S6a and b). In contrast, *C9* but not *C8* deletion blocked syncytia death (Fig. 1j). Further analysis showed that the cleavage of GSDME (N-GE) was abolished in *C9*^*−/−*^ cells (Fig. 1k). Interestingly, the S2’ fragment of SARS-CoV-2-S-Flag induced by cell-cell fusion was not observed in *C9*^−^^*/−*^ cells, indicating a linkage between caspase-9 and SARS-CoV-2 S protein cleavage (Fig. 1k and Supplementary Fig. S6c).

To further confirm that GSDME was involved in the death of syncytia, we generated GSDME knock-out (*GE*^*−/−*^-ACE2, *GE*^*−/−*^-SARS-CoV-2-S-Flag) and WT HeLa cell lines (*GE*^*+/+*^-ACE2, *GE*^*+/+*^-SARS-CoV-2-S-Flag), and co-cultured them. Similarly, GSDME did not affect cell fusion to form syncytia (Fig. 1l and Supplementary S6d and e), but GSDME knock-out significantly inhibited the death of syncytia (Fig. 1m). In addition, GSDME knock-out did not affect the activation of caspases (Fig. 1n), confirming it is downstream of caspases.

Our data indicated that the death of syncytia induced by SARS-CoV-2 infection could be mediated by GSDME-dependent pyroptosis. The existing transcriptomic data^13^ showed correlations of the expression between ACE2 and GSDME, especially in the small intestine and testis (Supplementary Fig. S7). The high-level expression of ACE2 and GSDME in testis could link to the destruction of male reproductive system by SARS-CoV-2 infection^14^. We analyzed single-cell-RNA-sequencing (scRNA-Seq) data from eight normal human lung transplant donors with a total of 42,225 cells^15^. As reported, the expression of ACE2 is concentrated in alveolar type 2 (AT2) cells, a special cell type with a small population in lung^16^ and GSDME is also enriched in AT2 cells (Supplementary Fig. S8a and b). Thus, GSDME-dependent pyroptosis could occur in SARS-CoV-2 infected AT2 cells.

Although cell death induced by SARS-CoV-2 infection has been shown by several studies^17,18^, in this study, we provide in vitro evidence showing that the syncytia formed by fusion of the cells expressing SARS-CoV-2 S protein and ACE2 respectively undergo pyroptosis (Fig. 1o). The pyroptosis is initiated by components of intrinsic apoptosis pathway and executed by caspase-3/7 mediated activation/cleavage of GSDME. Since scRNA-seq data showed that both ACE2 and GSDME were expressed in AT2 cells in human lung, we propose that GSDME-mediated syncytia death is a potential mechanism of the death of SARS-CoV-2 infection-caused syncytia. The lytic death of syncytia may contribute to the excessive inflammatory responses in severe COVID-19 patients.

## Supplementary information

Supplementary Figures and Methods

Supplementary Movie S1 The process of syncytia death recorded by the real-time observation system
