# Supplementary material for: Pyroptosis of syncytia formed by fusion of SARS-CoV-2 spike and ACE2-expressing cells
Source: Cell Discov. 2021 Aug 24;7:73. doi: 10.1038/s41421-021-00310-0 (PMC8384103; doi:10.1038/s41421-021-00310-0)
Supplement: Supplementary file 1 — Supplementary Figures and Methods [file 41421_2021_310_MOESM1_ESM.pdf]

Supplementary information for

Pyroptosis of syncytia formed by fusion of SARS-CoV-2 Spike and ACE2 expressing cells

Huabin Ma[1,2*], Zhoujie Zhu[1], Huaipeng Lin[2,3], Shanshan Wang[1], Peipei Zhang[2,3], Yanguo Li[1], Long Li[1], Jinling Wang[4], Yufen Zhao[1*], Jiahuai Han[2,3*]

This file includes:

Materials and Methods

Supplementary Figures S1 to S7, and legends

Supplementary Movie S1 title

References

Materials and Methods

Plasmids and cell lines

The plasmid of pCDNA6B-Flag-SARS-CoV-2-S provided by Prof. Peihui Wang (Shangdong University) was used as PCR template, then subcloned into pBOB-C-GFP, or pBOB-C-Flag lentivirus vector by the exonuclease III (Exo III)-assisted ligase-free cloning method as described previously[1]; ACE2 was amplified by PCR from our human cDNA library, and subcloned into pBOB lentivirus vector; The RFP-tagged organelle markers constructs were provided by Prof. Xin Chen (Xiamen University).

HeLa, A549, H1299, L929 and HEK293T were purchased from ATCC, and kept in Han lab at Xiamen university; All these cells were cultured in Dulbecco's modified Eagle's medium (DMEM), supplemented with 10% fetal bovine serum, 2 mM L-glutamine, 100 IU penicillin, and 100 mg/ml streptomycin and were kept at 37°C in a humidified atmosphere containing 5% CO2.

Antibodies and Reagents

The following antibodies were obtained from Proteintech: Caspase-8(13423-1-AP), Caspase-7(27155-1-AP), and ACE2(21115-1-AP); Cleaved-Caspase-3(9664) was purchased from Cell Signaling; Caspase-9(T55782), DYKDDDDK-Tag(3B9) were purchased from Abmart; Anti-DFNA5/GSDME antibody(ab215191) was purchased from Abcam.

Chloroquine (CQ) was obtained from MCE; The pan-caspase fmk inhibitor ZVAD was purchased from R&D Systems; Hoechst 33258 and LDH Cytotoxicity Assay Kit were purchased from beyotime; Cell Titer-Glo Luminescent Cell Viability Assay Kit was

obtained from Promega; Human IL-1β High Sensitivity ELISA Kit was obtained from MultiSciences (70-EK101BHS-96).

Generation of Knockout Cell Lines

C8$^{-/-}$, *C9*$^{-/-}$ and *GSDME*$^{-/-}$ cell lines were generated using CRISPR/Cas9 methods. The gRNA sites as followed:

Caspase-8： 5’-GCCTGGACTACATTCCGCAA-3’;

Caspase-9： 5’-CTGGCTTCACTCTTGCAAAG-3’;

GSDME: 5’- TTCTATGAGTACATCGCCAA-3’

The plasmids carrying the gRNA and Cas9 gene were packed into lentiviruses and then used to infect HeLa cells to mediate the gene editing. After puromycin (2ug/ml) selection, single clone was picked up. The disruption of target gene was determined by the sequencing of gene loci and by the immunoblot of cell lysates with antibodies.

Lentivirus Production and Infection

HEK293T cells were co-transfected with lentiviral vector carrying interested cDNA and lentivirus-packing plasmids (PMDL/REV/VSVG) by the calcium phosphate precipitation method, GFP as control; 8 hours later, the cell culture medium was replaced with fresh medium and the virus-containing medium was collected 48 h later, then centrifuged at 1000g for 3 min, kept at 4°C.

For infection, virus containing medium in the presence of 10 ug/ml Polybrene was added to cells plated in 12-well plates and then centrifuged at 1450g for 30 min at 37°C.The cell culture medium was replaced with fresh medium 8 h later. After 48h, the expression of indicated proteins were analyzed by western blot.

Confocal microscopy

All the images were obtained in live-cell scanning model. The cells harboring GFP or RFP tagged plasmid were cultured in 35mm Glass Bottom Culture Dishes (Nest). Imaging was carried out using the Zeiss LSM 780 with a 100x/1.49 NA oil objective in a 37°C incubator containing 5% CO_2. GFP was excited under a 488-nm argon laser, and RFP was excited under a 568-nm argon laser. Nuclei were stained using Hoechst 33342 (1:10,000) and excited under a 405-nm argon laser.

Western blot

The indicated cells were harvested and immediately lysed with 1.2x SDS sample buffer. Total cell lysates were separated by SDS-PAGE and transferred to polyvinylidene fluoride membranes (EMD Millipore, 0.22μm) for Western blot analysis with the appropriate antibodies. The proteins were visualized by enhanced chemiluminescence in accordance with the manufacturer's instructions (NcmECL Ultra, Enhanced Chemiluminescent, ECL).

Cell-cell fusion assay

ACE2-expressing cells and Spike-expressing cells were co-culture (2 x 10^5 cells/well cells mixed at a 1:1 ratio in 24-well plates); then images of 5 fields (20x objective lens) were taken at indicated time by ZEISS Axio Observer; Nucleus was stained by Hoechst, counted by image J. The fusion index (FI) was calculated as "% of nuclei in fused cells"[2].

Analysis of scRNA-seq data

We analyzed a dataset (Gene Expression Omnibus GSE122960) freely available from

public database. We used the R (version 3.6.3) and Seurat R package (version 3.9.9.9010). Firstly, Low-quality cells with less than 200 detected genes and genes found to be expressed in less than three cells were removed. Then, according to the median number of genes and the percentage of mitochondrial genes in the lung samples, we filtered cells that have unique feature counts over 5000 and cells that have >10% mitochondrial counts. After QC (Quality control), 42225 high quality lung cells were obtained.

For each individual sample, we employed a global-scaling normalization method "LogNormalize" that normalizes the feature expression measurements for each cell by the total expression, multiplies this by a scale factor (10,000 by default), and log-transforms the result. Identification of highly variable features was implemented in the FindVariableFeatures function. We detected the batch effect between eight different lung samples. So, we used the functions (FindIntegrationAnchors and IntegrateData) to integrate all eight individual samples and mitigate batch effects. Mitochondrial genes and differences in cell cycle stages between proliferating cells were regressed out by specifying the vars.to.regress argument in Seurat function ScaleData. We performed PCA on the scaled data, only the previously determined variable features were used as input. Identification of the true dimensionality of the dataset for further analysis was constructed by generating an "Elbow plot".

To cluster the cells, we first used the FindNeighbors function to construct a KNN graph based on the euclidean distance in PCA space, and refine the edge weights between any two cells based on the shared overlap in their local neighborhoods. We next iteratively

grouped cells together by using FindClusters function in Seurat. With a resolution of 0.2, cells were clustered and classified into 18 different cell types. The process to identify the cluster marker genes was implemented by the function FindAllMarkers in Seurat. Referring to three databases (CellMarker; PanglaoDB-A Single Cell Sequencing Resource For Gene Expression Data; The Human Protein Atlas), cell type assignment was performed based on the marker genes). We visualized the dataset with tSNE (t-distributed stochastic neighbor embedding) a non-linear dimensional reduction technique.

Supplementary Figures

Fig. S1

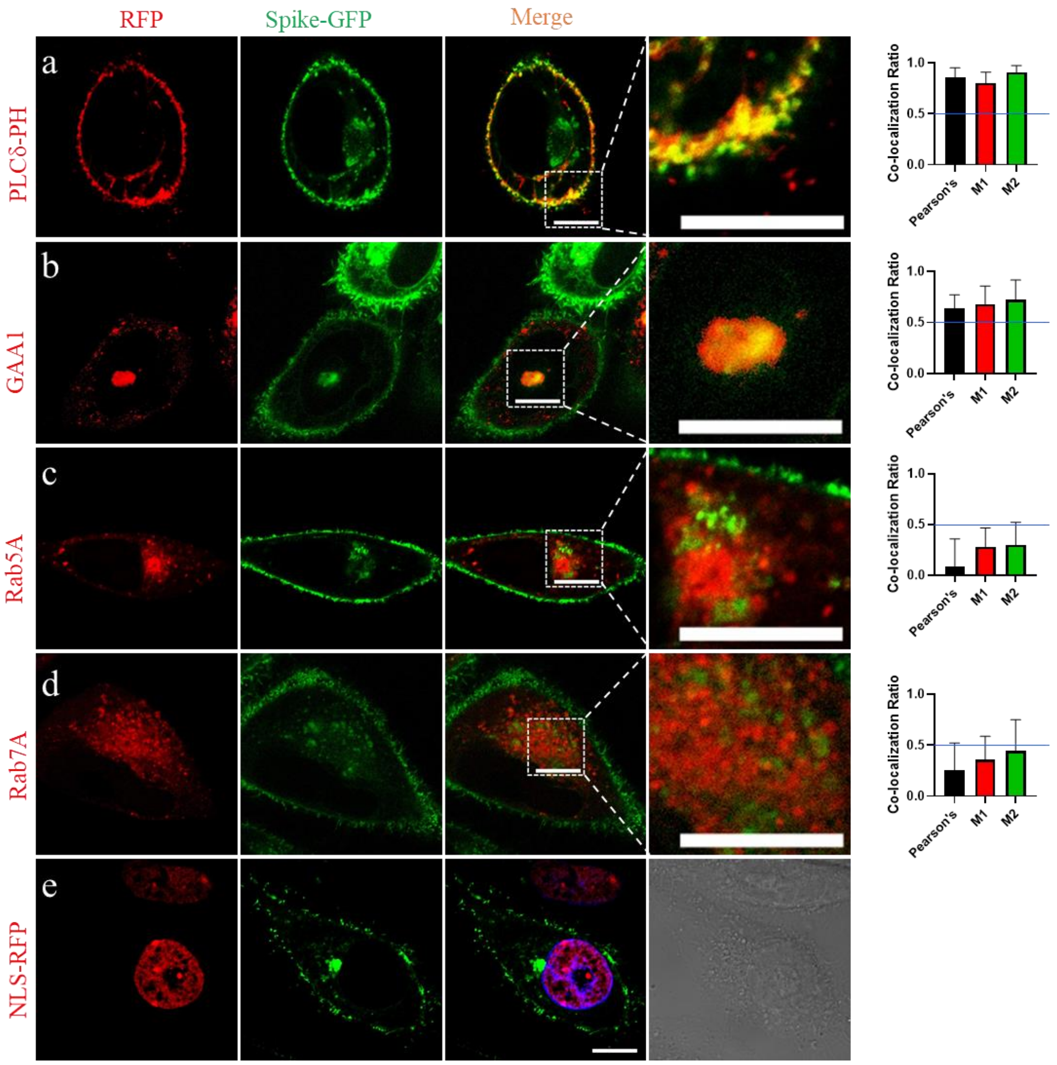

Fig. S1 Cellular distribution of SARS-CoV-2-S-GFP.

Co-expression of SARS-CoV-2-S-GFP with different markers fused with RFP in HeLa cells: (**a**). PLCδ-PH is the cell membrane marker; (**b**). GGA1 is a Golgi associated protein; (**c**). Rab5A is mainly localized in early endosome membrane or another cytosolic vesicle; (**d**). Rab7A governs early-to-late endosomal maturation; (**e**). NLS-RPF was referred as insert the nuclear localization sequence (NLS) of SV40 at the head of RPF. The images were scanned by LSM780, the nucleus(blue) was stained by

Hoechst; Bar, 20 μm.

For colocalization analysis, all image were selected and analyzed using the JACoP plugin of ImageJ software. Pearson's correlation coefficient (PCC) and Manders' Colocalization Coefficients (MCC) were used to quantify colocalization. Pearson's correlation coefficient (Rr): -1.0 - 1.0; Values indicating colocalization: 0.5 -1.0; Values indicating absence of colocalization: -1.0 -0.5; Manders' Colocalization Coefficients (MCC): M1 and M2: vary; Values indicating colocalization:>0.5; Values indicating absence of colocalization: <0.5[3,4].

Fig. S2

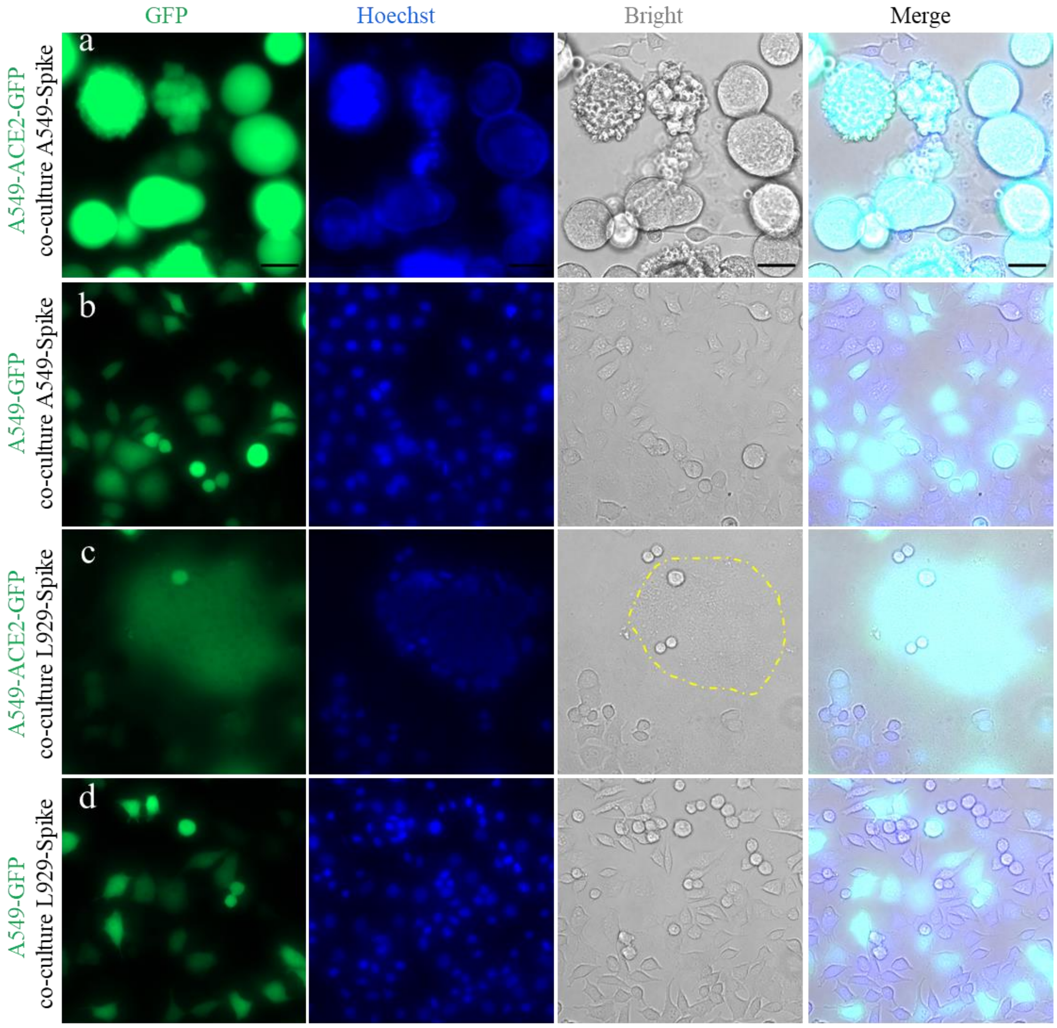

Fig. S2 Syncytia formation was dependent on the expression of Spike and ACE2

A549-ACE2-GFP cells was co-cultured with A549-Spike (or L929-Spike) cells (**a** and **c**), and A549-GFP cells was co-cultured with A549-Spike (or L929-Spike) cells (**b** and **d**), four hours later, syncytia formation in both ACE2 and Spike-expressing cells, nucleus was stained by Hoechst, bar, 50 μm.

Fig. S3

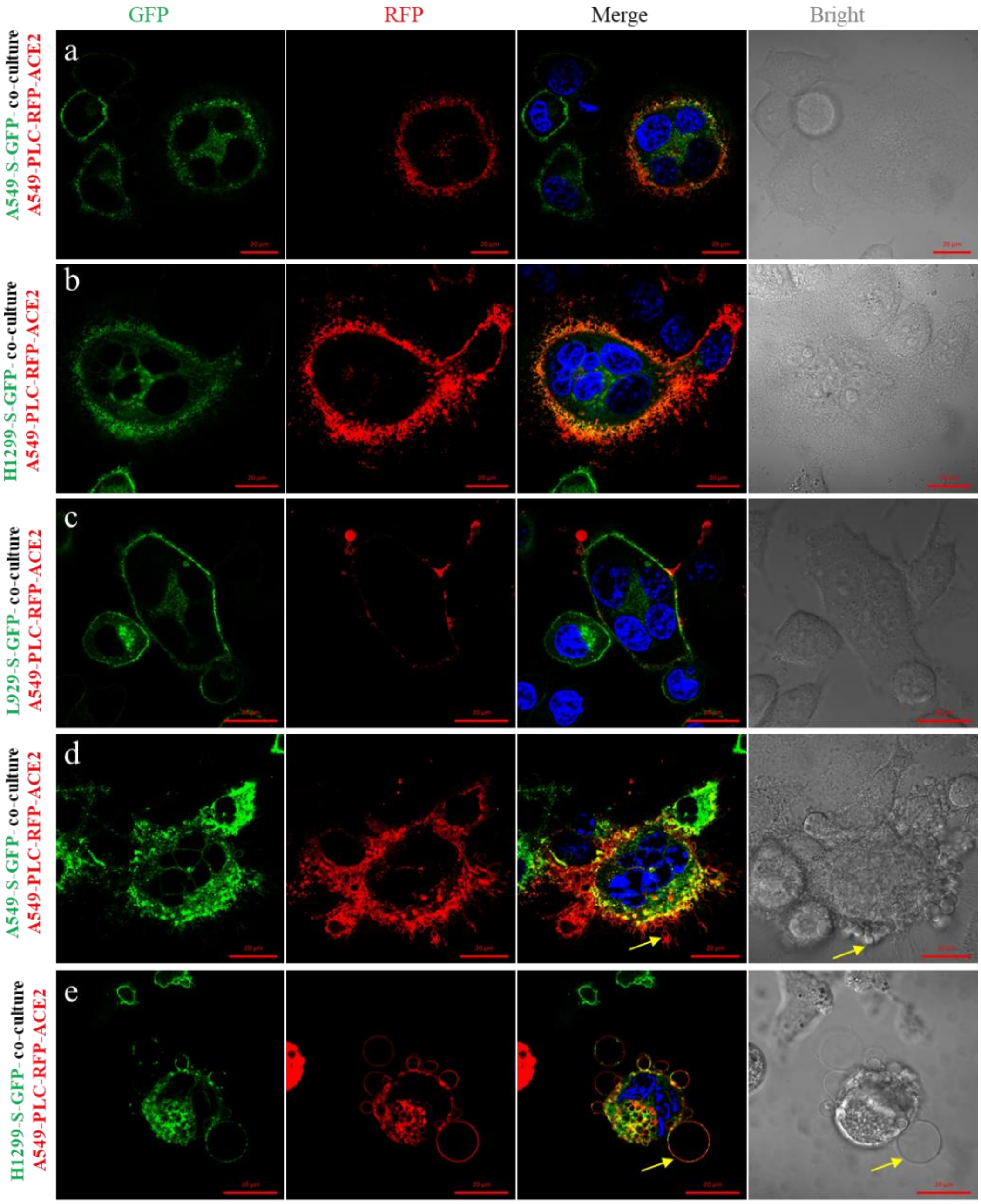

Fig. S3 related to Fig. 1a

PLC-RFP-A549-ACE2 cells were respectively co-cultured with SARS-CoV-2-S-GFP-A549 cells, SARS-CoV-2-S-GFP-H1299 cells, SARS-CoV-2-S-GFP-L929 cells at a 1:1 ratio, 4 hours later (**a, b, c**), or 12 hours later (**d** and **e**), image was scanned by LSM780, the nucleus(blue) was stained by Hoechst; Bar, 20 μm.

Fig. S4

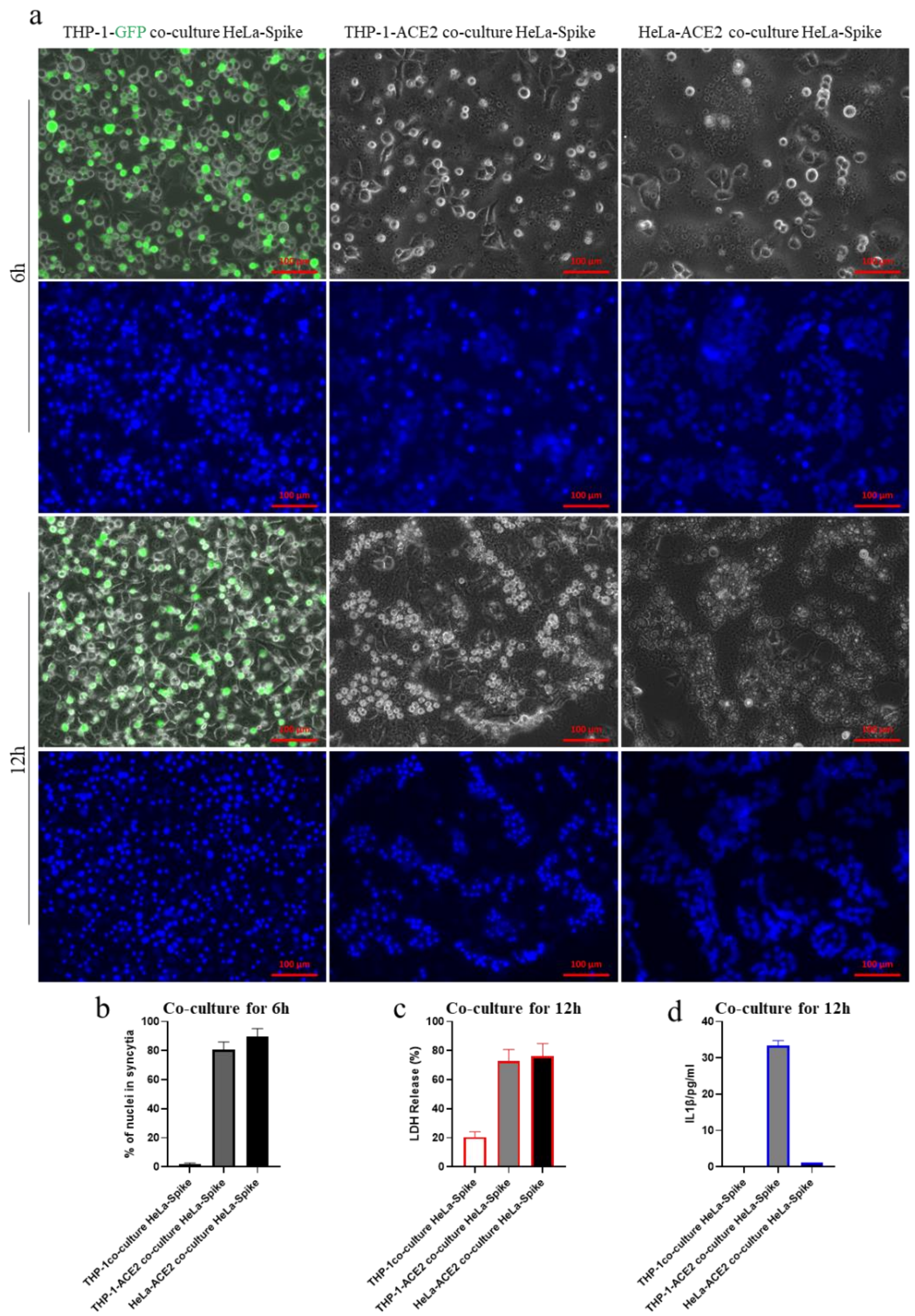

Fig. S4 related to Fig. 1e to j

THP-1-GFP, THP-1-ACE2, HeLa-ACE2 cells were co-cultured with HeLa-Spike cells respectively. Syncytia formation was observed at 6h (**a** and **b**). cell death and IL1β release were detected at 12h (**c** and **d**).

Fig. S5

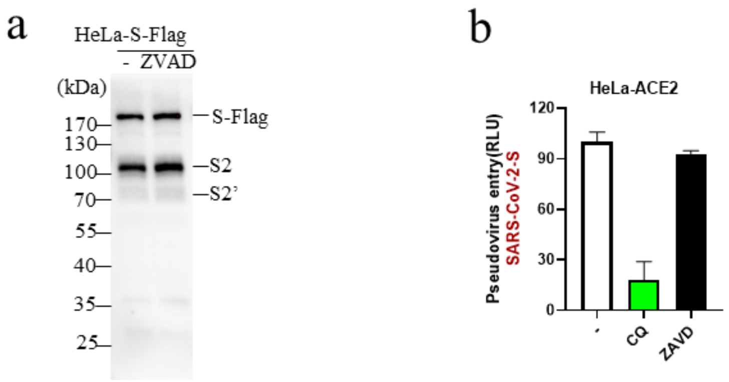

Fig. S5 related to Fig. 1h

ZVAD treatment did not affect S protein priming (**a**), ZVAD treatment also failed to inhibit the SARS-CoV-2-Spike pesudovirus entry HeLa-ACE2 cells, chloroquine (CQ) [5]was the positive control which inhibited the SARS-CoV-2-Spike pesudovirus entry (**b**).

Fig. S6

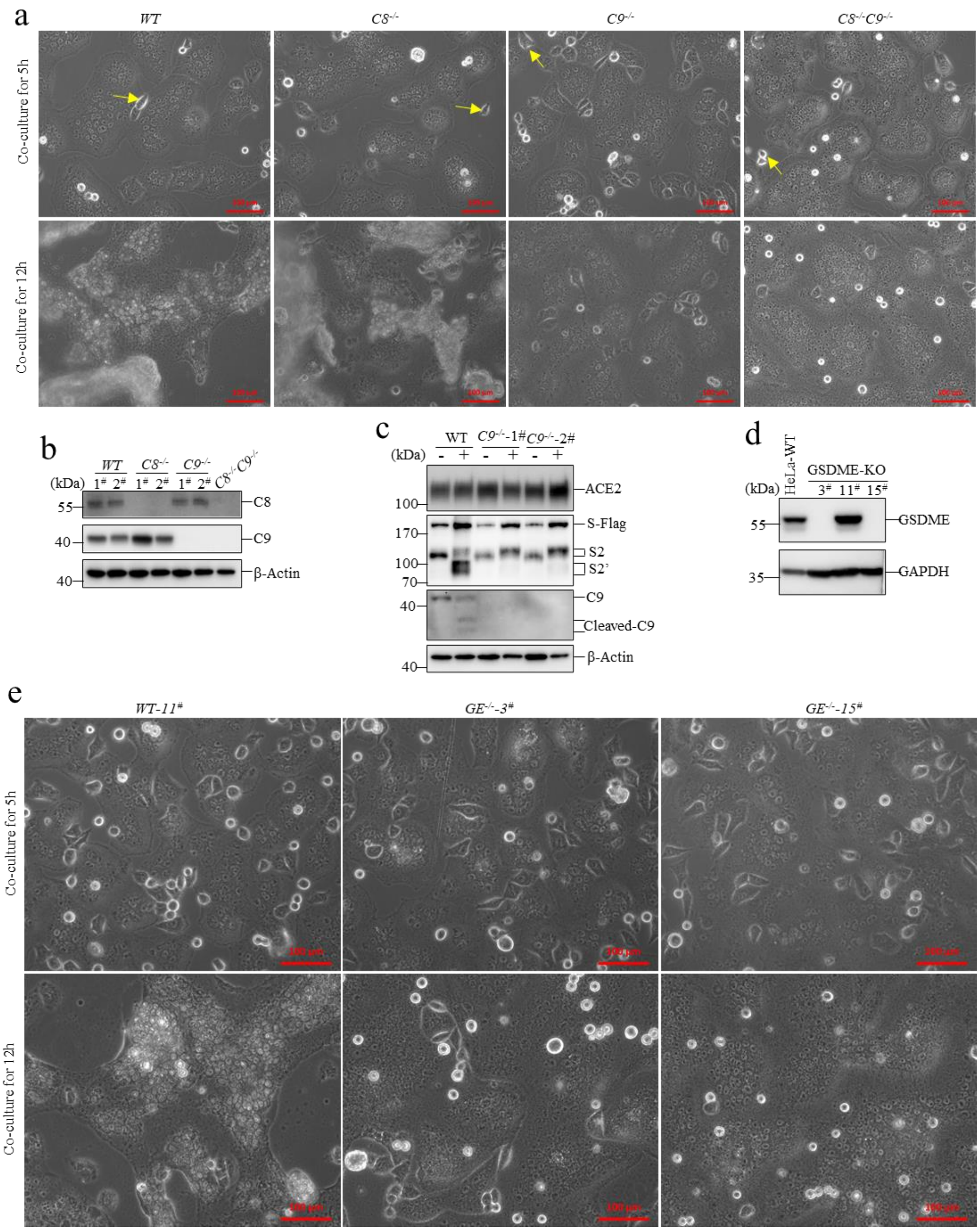

Fig. S6 related to Fig. 1i-n

a. Generated Caspase-8(*C8*$^{-/-}$), Caspase-9(*C9*$^{-/-}$) knock-out, Caspase-8 and Caspase-9 double knock-out (*C8*$^{-/-}$*C9*$^{-/-}$) HeLa cells by CRISPR-Cas9; then transfected these cells with ACE2, or SARS-CoV-2-S-Flag to obtained wild-type HeLa (*WT*-ACE2; *WT*-S-Flag); Caspase-8 knock-out (*C8*$^{-/-}$-ACE2; *Casp8*$^{-/-}$-S-Flag);

Caspase-9 knock-out (*Casp9*$^{-/-}$-ACE2; *Casp9*$^{-/-}$-S-Flag); Caspase-8/9 double knock-out (*C8*$^{-/-}$*C9*$^{-/-}$-ACE2; *C8*$^{-/-}$*C9*$^{-/-}$-S-Flag) cells; then co-culture these cells respectively, images were obtained by Axio Observer at 5 hours indicated for syncytia formation, and 12 hours for cell death; Bar, 100 μm.

b. Western blot analyzed these knock-out cell clones.

c. To exclude the clonal effect, another individual clone was used to repeat the experiments, which showed the cleavage of Spike (refer to S2') induced by syncytia formation disappeared in C9-deficient cells.

d. Generated GSDME knock-out HeLa cells by CRISPR-Cas9, western blot analyzed the GSDME-KO cell lines.

e. Transfected GSDME cells with ACE2, or SARS-CoV-2-S-Flag to obtained wild-type HeLa (*WT-11*$^{\#}$-ACE2; *WT-11*$^{\#}$-S-Flag); GSDEM knock-out (*GE*$^{-/-}$-3/15$^{\#}$-ACE2; *GE*$^{-/-}$-3/15$^{\#}$- S-Flag) cells; then co-culture these cells respectively, images were obtained by Axio Observer at 5 hours indicated for syncytia formation, and 12 hours for cell death; Bar, 100 μm.

Fig. S7

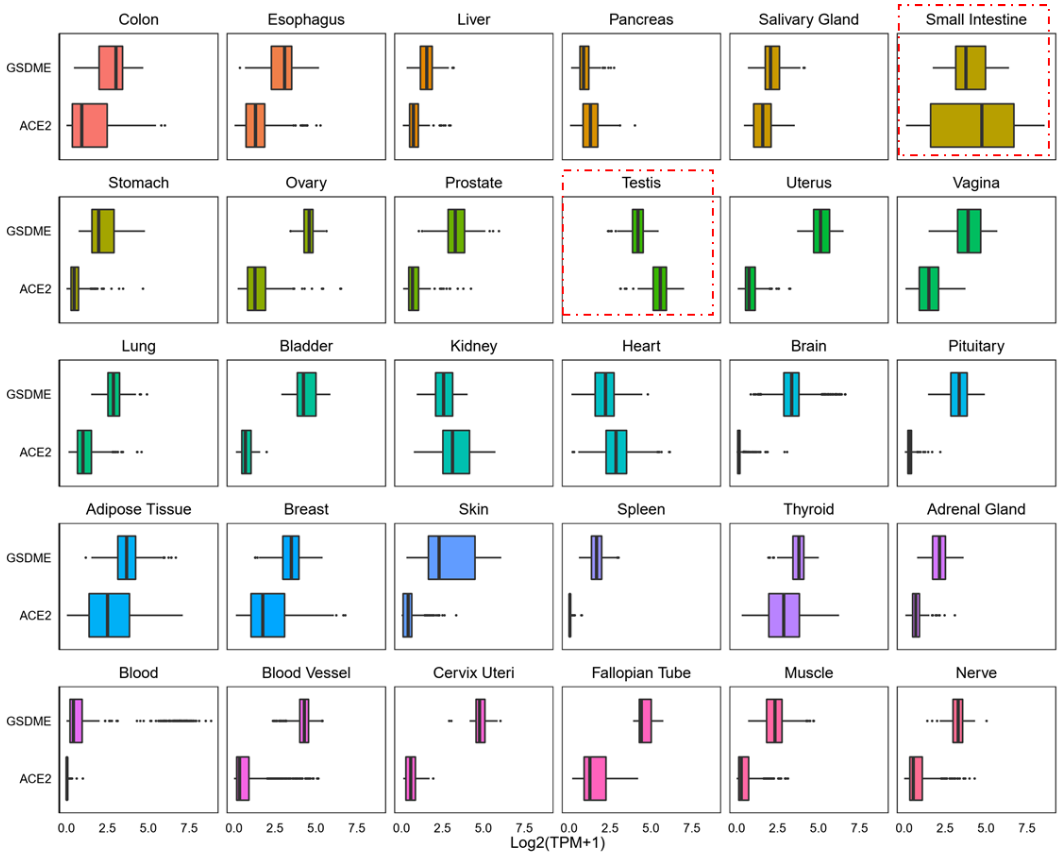

Fig. S7 Expression profile of GSDME and ACE2.

Data from the Genotype-Tissue Expression (GTEx) Project for tissues.

Fig. S8

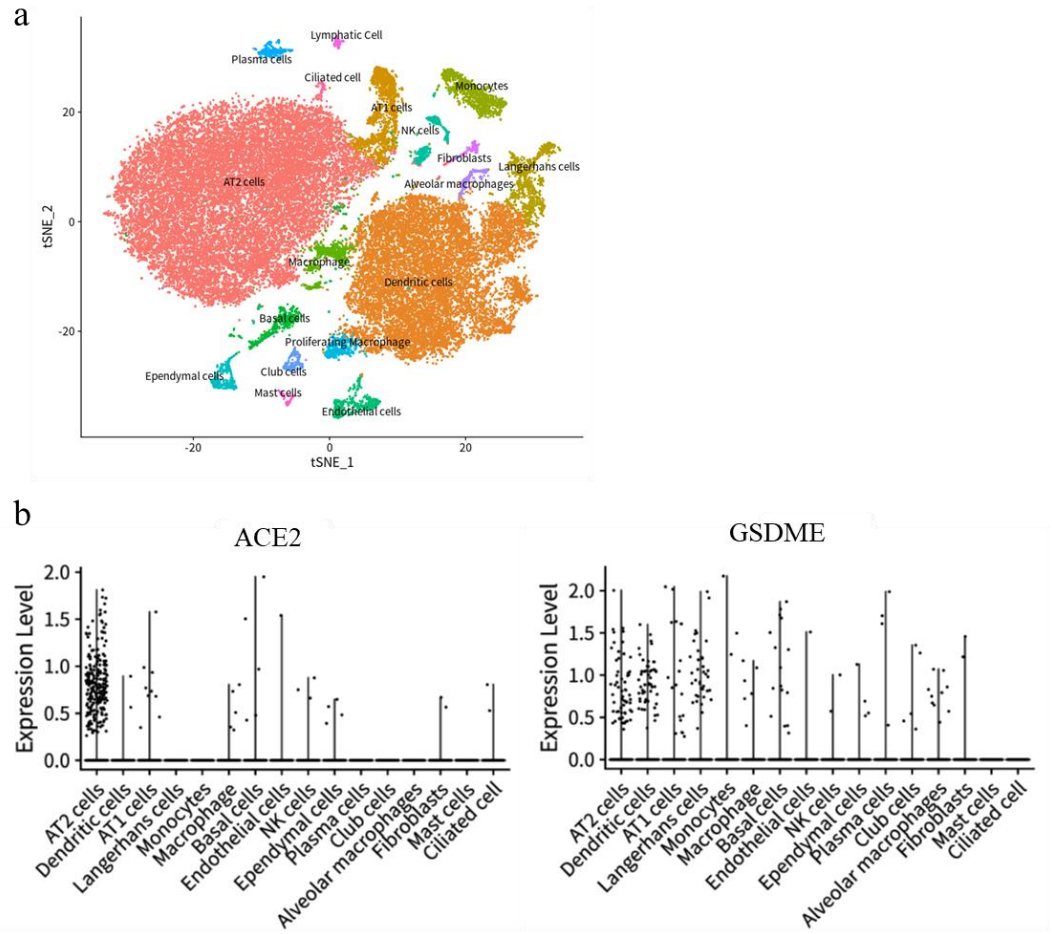

Fig. S8 scRNA-seq profile of GSDME and ACE2 in human lung

a. Cellular populations identified. Cells were clustered using a graph-based shared nearest neighbor clustering approach and visualized using a t-distributed Stochastic Neighbor Embedding (tSNE) plot. AT1 = alveolar type I; AT2 = alveolar type II.

b. Violin plots representing expression probability distributions across clusters.

Supplementary Movie S1

The process of syncytia death recorded by the real-time observation system

HeLa-ACE2 cells were co-cultured with HeLa-Spike cells, then, the fate of syncytia was recorded every 30mins by the real-time observation system (Leica Flyer Paula), bar, 100 μm.

References:

1 Ma, H. *et al.* Inactivation of Cyclic AMP Response Element Transcription Caused by Constitutive p38 Activation Is Mediated by Hyperphosphorylation-Dependent CRTC2 Nucleocytoplasmic Transport. *Molecular and cellular biology* **39**, doi:10.1128/mcb.00554-18 (2019).

2 Zhang, Z. *et al.* SARS-CoV-2 spike protein dictates syncytium-mediated lymphocyte elimination. *Cell Death & Differentiation*, doi:10.1038/s41418-021-00782-3 (2021).

3 Dunn, K. W., Kamocka, M. M. & McDonald, J. H. A practical guide to evaluating colocalization in biological microscopy. *American journal of physiology. Cell physiology* **300**, C723-742, doi:10.1152/ajpcell.00462.2010 (2011).

4 Zinchuk, V. & Grossenbacher-Zinchuk, O. Recent advances in quantitative colocalization analysis: focus on neuroscience. *Progress in histochemistry and cytochemistry* **44**, 125-172, doi:10.1016/j.proghi.2009.03.001 (2009).

5 Wang, M. *et al.* Remdesivir and chloroquine effectively inhibit the recently emerged novel coronavirus (2019-nCoV) in vitro. *Cell Res* **30**, 269-271, doi:10.1038/s41422-020-0282-0 (2020).
